# Molecular Identification of *Eimeria* Species in Broiler Chickens in Trinidad, West Indies

**DOI:** 10.3390/vetsci5010012

**Published:** 2018-01-22

**Authors:** Arianne Brown Jordan, Damer Blake, Jamila Beard, Asha Beharry, Louanne Serrette, Atlyn Soleyn, Jamie Sookhoo, Lemar Blake, Gabriel Brown, Christopher Oura

**Affiliations:** 1 Department of Basic Veterinary Sciences, School of Veterinary Medicine, The University of the West Indies (St. Augustine), Eric Williams Medical Sciences Complex, Mount Hope, Republic of Trinidad and Tobago; arianne.brown@my.uwi.edu (A.B.J.); Jamie.sookhoo@sta.uwi.edu (J.S.); Lemar.Blake@sta.uwi.edu (L.B.); 2 Department of Pathobiology and Population Sciences, The Royal Veterinary College, Hertfordshire AL9 7TA, UK; dblake@rvc.ac.uk; 3 School of Veterinary Medicine, The University of the West Indies (St. Augustine), Eric Williams Medical Sciences Complex, Mount Hope, Republic of Trinidad and Tobago; jamilabeard@gmail.com (J.B.); beharryasha@gmail.com (A.B.); louanne.serrette@gmail.com (L.S.); atlynsoleyn@gmail.com (A.S.); 4 Department of Veterinary Clinical Sciences, School of Veterinary Medicine, The University of the West Indies (St. Augustine), Eric Williams Medical Sciences Complex, Mount Hope, Republic of Trinidad and Tobago; gabriel.brown@sta.uwi.edu

**Keywords:** coccidiosis, coccidia, *Eimeria*, poultry, Trinidad

## Abstract

Coccidiosis is an intestinal disease of chickens of major economic importance to broiler industries worldwide. Species of coccidia found in chickens include *Eimeria acervulina*, *Eimeria brunetti, Eimeria maxima, Eimeria mitis, Eimeria necatrix, Eimeria praecox,* and *Eimeria tenella*. In recent years, polymerase chain reaction (PCR) has been developed to provide accurate and rapid identification of the seven known *Eimeria* species of chickens. The aim of this study was to use species-specific real-time PCR (qPCR) to identify which of the seven *Eimeria* species are present in Trinidad poultry. Seventeen pooled fecal samples were collected from 6 broiler farms (2–5 pens per farm) across Trinidad. Feces were also collected from birds showing clinical signs of coccidiosis in two live bird markets (pluck shops). qPCR revealed the presence of five species of *Eimeria* (*E. acervulina, E. maxima, E. mitis, E. necatrix*, and *E. tenella*)*,* but not *E. brunetti* or *E. praecox*. Mixed infections were detected on all broiler farms, and DNA of two highly pathogenic *Eimeria* species (*E. tenella* and *E.* *necatrix*) was detected in feces taken from clinically sick birds sampled from the two pluck shops.

## 1. Introduction

Coccidiosis is one of the most important and costly diseases affecting domestic chickens worldwide and is of major economic importance to the broiler industry in Trinidad and Tobago (T&T) and the wider Caribbean. Estimates are that the annual costs incurred by coccidiosis to the poultry industry exceed US$2.5 billion worldwide [1]. Through a combination of parasite ubiquity, fecundity, and pathogenicity, coccidiosis is one of the top ten veterinary diseases, having a detrimental impact on resource limited parts of the developing world. It is also one of the ten most economically significant endemic livestock diseases in the developed world [2]. This disease can inflict severe damage to the host’s intestine, commonly causing increased morbidity and mortality [3]. One of the challenges leading to its economic importance is the difficulty in diagnosing cases of subclinical infection, which cause increased feed conversion ratios and failure to thrive [4]. Sub-clinical infection can also predispose towards conditions such as necrotic enteritis [5].

Seven *Eimeria* species are recognized as infecting chickens; these species exhibit variable levels of pathogenicity. *E. necatrix* and *E. tenella* are considered to be the most pathogenic, causing intestinal hemorrhage as well as high morbidity and mortality in naive chickens [6,7]; *E. acervulina*, *E. brunetti*, and *E. maxima* can cause clinical disease, whereas *E. mitis* and *E. praecox* are thought to be fairly non-pathogenic [7] but can cause increased feed conversion ratios and reduced growth rates [8].

Classical methods for the identification of *Eimeria* species are challenging and time-consuming and require highly trained personnel [9,10]. These methods include observation of clinical signs in the infected animals, macroscopic lesions on necropsy, and parasite biology and morphological characteristics of the oocysts. Identification through these methods sometimes lacks accuracy due to similarities in characteristics across species and because mixed infections are common under field conditions [10]. In recent years, however, quantitative real-time polymerase chain reaction (qPCR) assays have been developed that provide a faster and more reliable method for the detection and quantification of the seven *Eimeria* species which infect chickens [3].

The poultry industry is the biggest of the livestock industries in Trinidad and Tobago (T&T), with around 32 million chickens and 556 broiler farms (T&T Central Statistical Office, 2010). *Eimeria* parasites pose a significant threat to this industry affecting poultry health and welfare, production, food security, and economics. To date, however, little or no information has been published on the prevalence, incidence, identity, and impact of *Eimeria* parasites within T&T and the wider Caribbean region. The aim of this study was to fill this knowledge gap by using molecular qPCR tools to identify which *Eimeria* species are present within Trinidadian poultry production systems and to elucidate what risks they pose. Knowledge defining pathogen occurrence in T&T is essential for the development of effective coccidiosis control programs on local broiler farms.

## 2. Materials and Methods 

### 2.1. Study Flocks

A total of six independently operated broiler farms contracted to grow birds for a single broiler producing company that provide day-old chicks, feed, vaccines, and veterinary care, were selected for sampling. Farm selection was convenience based upon gaining permission to sample from the overarching broiler producing company, as well as the individual farmers. All individual pens, housing isolated batches of birds between 4 and 6 weeks of age were sampled on each farm. This amounted to a total of 17 pens from 6 farms: Farm 1 (3 pens), Farm 2 (5 pens), Farm 3 (2 pens), Farm 4 (2 pens), Farm 5 (2 pens), and Farm 6 (3 pens).

Relevant information was collected including the date, type of sample, type of production unit, age of flock, along with details of health status and any recent treatment for coccidiosis. Based on gathered information, some batches of the birds on the farms have experienced sporadic clinical signs consistent with coccidiosis, but no evidence of the disease was observed on any of the broiler farms at the time of sampling. The six farms sampled in this study routinely fed their birds with the same regimen of in-feed coccidiostat drug salinomycin in starter, grower, and finisher feeds, which were all obtained from the same broiler producing company. The broiler farms all had conventional, open-sided, earthen-floored pens with uninsulated, metal roofs. Bell drinkers and manual feeding systems provided drinking water and feed for the chickens. Wood shavings mixed with some sawdust were used as litter on all farms. Several factors including litter and water management, water quality, roof condition, and pen design and orientation, could have influenced the litter condition in each pen. As such, pooled samples from individual pens were used as experimental units.

Two live bird markets (otherwise known as pluck shops), where birds are temporarily housed prior to slaughter, were also sampled. Pluck shop house broilers ready for consumption, so coccidiostats are not given at these locations. The coccidiostat treatment regimens carried out on the originating farms prior to arriving at the pluck shops were not available, as it was not possible to trace the sampled birds back to their original location.

### 2.2. Sample Collection and Processing

Falcon tubes (50 mL) were filled with 30 mL of 2% potassium dichromate (K_2_Cr_2_O_7_). Using a clean, wooden tongue depressor, fecal matter was collected to fill the tube to the 50 mL mark, while walking in a “zig-zag” pattern, wall to wall, in each of the 17 pens sampled. Two (2) tubes were collected from each pen; each tube was collected from different starting points. The tubes were then capped, gently inverted five times, sealed with parafilm, and labeled for transport to the laboratory in sealed bags. At the laboratory, the contents of each falcon tube were emptied into a correspondingly labeled, clean, sterile cup. The cups were covered with a perforated sheet of parafilm, and left to stand at room temperature for three days to achieve some degree of sporulation. The samples were then returned to their respective falcon tubes, which were placed in sealed bags, and refrigerated at 4 °C.

All sampling procedures were carried out with formal approval in full respect of international legal and ethical requirements and code of practice enforced by The University of the West Indies and the nation of Trinidad and Tobago for research in animals.

### 2.3. Recovery and Purification of Genomic DNA 

Genomic DNA was extracted from the oocysts as described previously [11]. Essentially, oocysts were washed in TE buffer and were disrupted in a Mini-BeadBeater-16 (BioSpec, Bartlesville, OK, USA) using 2 mL of sample and sterile No. 8 glass beads (0.4–0.6 mm diameter range) before the DNA was extracted from the lysate using a QlAamp Stool Mini Kit (Qiagen, Hilden, Germany). DNA extraction was carried out in duplicate for each sample.

### 2.4. Real-Time Polymerase Chain Reaction (qPCR) Amplification

Using an ABI 7500 Applied Biosystem PCR machine, qPCR was carried out for each of the seven *Eimeria* species using the primers and probes, as well as the protocol, previously described [3]. The TaqMan^®^ probes were labeled with 6-carboxyfluorescein (6-FAMTM) at the 5′ end and with TAMRA quencher at the 3′ end. Each species-specific assay was initially validated using previously purified control DNA representing reference strains of each *Eimeria* species (Houghton strain for all with the exception of *E. maxima*, which was extracted from the Weybridge strain) [12]. qPCR assays for all seven *Eimeria* species, along with positive and negative controls (RVC, London, UK), were run in duplicate for each sample. The cycle threshold for each *Eimeria* species was set based on positive controls of known Ct values. The total number of amplification cycles was 50. The estimated amounts of target DNA, in the form of a Ct value, was obtained for the study samples.

## 3. Results

Out of the seven known *Eimeria* species, DNA representing four (*E. acervulina, E. maxima, E. mitis* and *E. tenella*) were detected in the fecal samples from broiler farms. DNA representing a fifth species (*E. necatrix*) was detected in feces from birds showing clinical signs of coccidiosis in one of the two pluck shops that were sampled. The two remaining *Eimeria* species (*E. brunetti* and *E. praecox*) were not detected in feces from any of the sampled pens.

*Eimeria* spp. DNA was found to be present in the pooled fecal samples collected from all 17 pens that were sampled on the six poultry farms (Table 1). Overall, the most prevalent species was *E. tenella,* which was detected in all 17 sampled pens from the 6 farms. The second most prevalent species was *E. acervulina* which was present in all the pens sampled on Farms 1, 2, 3, 4, and 6, but was not detected in the two pens sampled on Farm 5. *E. maxima* was detected in the majority of pens sampled on Farms 1, 3, 4, 5, and 6, but was not detected in the five pens that were sampled on Farm 2. *E. mitis* was only detected at very low levels (Ct values > 37) in one pen on each of Farms 2, 5, and 6.

Mixed infections with two or more *Eimeria* species were found in all 17 pens from the six farms (Table 2). The most prevalent combination (47% of all combinations) was *E. acervulina*, *E. maxima*, and *E. tenella*, which were present in eight pens on four of the farms. Five out of 17 pens were found to be co-infected with *E. acervulina* and *E. tenella* and one pen on Farm 6 was co-infected with four *Eimeria* species (*E. acervulina*, *E. maxima, E. mitis,* and *E. tenella).* Other combinations of infection found were *E. acervulina*, *mitis,* and *tenella* and *E. mitis* and *tenella*. Over all the pens sampled, the average complexity of infection (% species detected per farm) was 43% for Farms 1–4, 29% for Farm 5, and 57% for Farm 6. In Pluck Shop 1, the complexity of infection was 43%, while it was 29% in Pluck Shop 2.

High levels of DNA for *E. tenella* (Ct = 27.3) and *E. acervulina* (Ct = 28.4) were detected in the pooled fecal samples collected from one of the two pluck shops that were sampled (Pluck Shop 1). This sample also contained lower levels of DNA for *E. maxima* (Ct = 33.7) and *E. mitis* (Ct = 36.6). Interestingly, pooled fecal samples taken from the second pluck shop (Pluck Shop 2) contained relatively high levels of DNA for *E. necatrix* (Ct = 29.9) and low levels of DNA for *E. acervulina* (Ct = 38.2). This was the only sample in the study in which *E. necatrix* was detected (Table 1).

## 4. Discussion

Species-specific qPCR was successfully used to determine that five of the seven recognized *Eimeria* species, which infect chickens, were circulating in Trinidad broilers. Four of the identified species (*E. acervulina, E. maxima, E. necatrix*, and *E. tenella*) are considered to be pathogenic or highly pathogenic in poultry. Although *E. mitis* is not considered to be highly pathogenic, it has been associated with increased feed conversion ratios as well as morbidity [8] and therefore has the potential to cause economically significant reductions in chicken productivity.

The qPCR performed in this study was semi- quantitative rather than fully quantitative, adopted to streamline the diagnostic process by removing the requirement for gel electrophoresis. Although we were unable to measure the precise gene copy numbers through comparison with a standard curve, we were able to compare Ct values between the samples. Considering that each cycle there is a “doubling” of amplicons (assuming 100% efficiency), three Cts are equivalent to an eight-fold difference in copy numbers. Ct values of around 29 (as seen in the samples containing *E. necatrix* and *E. tenella* from the two pluck shops would be expected to contain around 60 times more *Eimeria* DNA than samples with Ct values of around 35, and around 500 times more DNA that samples with Ct values of around 38, as seen in many of the samples from the broiler farms. In this way, it was possible to compare the relative amounts of *Eimeria* DNA that were present in the samples on a scale of high (Ct > 30), moderate (Ct 30–35), and low (Ct < 35), although it was not possible to measure the exact amount of gene copies per gram of feces. The semi-quantitative nature of the qPCR used in this study therefore has clear advantages over the use of conventional PCR.

It is important to note, however, that moderate to low amounts of parasite DNA were identified in the majority of fecal samples collected from the broiler farms (Ct > 30), and no clinical signs of *Eimeria* infection were observed in the birds at the time of sampling. Farmers did however report that some clinical signs of coccidiosis had occasionally been observed in previous batches of birds on their farms in the past. The birds in this study were sampled at 4–6 weeks of age, the peak time of oocyst shedding and accumulation in the litter [13]. Further, the most widely used anticoccidial drugs (the ionophores), which were used on the broiler farms in this study, have been recognized to exert incomplete anticoccidial control, even in apparently naïve parasite populations, permitting the induction of protective immunity which can be of value during pre-slaughter drug withdrawal periods [14]. Thus, the absence of clinical disease despite the presence of parasite DNA on the sampled farms suggests that the current control methods employed, utilizing routine in-feed anticoccidial prophylaxis, provide incomplete but sufficient protection under current broiler management practices. However, further investigation is needed to examine possible anticoccidial drug resistance and the economic consequences of sub-clinical infection.

Sub-clinical infection can lead to severe economic losses from reduced weight gain and increased feed conversion ratios in affected birds. Intensive chicken farming across the world, including in T&T, depends on specific prophylaxis to control coccidiosis with in-feed anticoccidial drugs and, in some markets, live vaccines [1]. It has been demonstrated that, over time, anticoccidial drugs have become less effective due to the development of drug resistance [15]. Drug-resistant *Eimeria* strains are responsible for sub-clinical coccidiosis and resultantly for reduced broiler performance [16]. Interestingly, the economic importance of sub-clinical coccidiosis varies with the composition and dynamics of coccidial populations [17]. For that reason, the identification and genetic characterization of the various *Eimeria* species that are circulating in a particular chicken population is essential to coccidiosis prevention and control efforts [18]. Extensive studies on more birds from different management and production systems (layer, broiler, and backyard), including anticoccidial resistance studies, are required in order to better understand the economic impact of coccidiosis on the poultry industry of T&T.

In fecal samples collected from the two sampled pluck shops, where mild clinical signs of coccidiosis were observed in the birds, high levels of *Eimeria* DNA (Ct ≤ 30) were detected for *E. acervulina* and *E. tenella* in the first pluck shop, and *E. necatrix* in the samples taken from the second pluck shop. It is therefore possible that the clinical signs observed in the birds from these two pluck shops may have been caused by a combination of *E. acervulina* and *E. tenella* in the first pluck shop and *E. necatrix* in the second pluck shop. Most pluck shops in Trinidad largely kill and market birds obtained from farms contracted by any of four large broiler producing companies and from smaller backyard independent poultry farms. These four large broiler producers treat their birds with different regimens of in-feed anticoccidial products (e.g., the ionophores salinomycin or monensin) and when necessary treat clinical coccidiosis with sulfonamides or amprolium in the drinking water. The smaller backyard poultry farms, however, conduct less rigid coccidiostat treatment regimes, in some cases using no treatments. It was not possible to trace the sampled birds from the pluck shops to their farms of origin, but it is possible that the clinical signs observed may have been a result of reduced levels of, or different types of, coccidiostats used on the origin farms.

This study highlights the presence of five species of *Eimeria*, four of which are considered to be pathogenic, circulating in Trinidad broiler chickens. Since the only method of coccidiosis transmission is for a bird to ingest sporulated oocysts and all local broilers are reared on the floor, access to and ingestion of oocysts from potentially pathogenic *Eimeria* species, by chickens in the pen, cannot be prevented. Because vaccination is not currently used for coccidiosis prevention in local broiler production, the findings of this study therefore emphasize the importance of maintaining effective chemoprophylactic control strategies. This fundamental knowledge will impact the success of future anticoccidial control strategies and will be of crucial value in selecting the most efficacious anticoccidial vaccine candidates for use within T&T. The data presented here, though limited to identification of *Eimeria* species, is important and useful baseline data that will enable further studies to be carried out on *Eimeria* prevalence, leading to improved treatment, control, and prevention strategies in the region.

## Figures and Tables

**Table 1:** Real-time polymerase chain reaction (qPCR) results for the seven (7) Eimeria species of poultry in pooled fecal samples taken from multiple pens on six broiler farms and two pluck shops in Trinidad, West Indies.

Farm	Pen Number	*E. acervulina* (Mean Ct)	*E. brunetti* (Mean Ct)	*E. maxima* (Mean Ct)	*E. mitis* (Mean Ct)	*E. necatrix* (Mean Ct)	*E. praecox* (Mean Ct)	*E. tenella* (Mean Ct)
Farm 1	Pen 1	37.1	No Ct	37.6	No Ct	No Ct	No Ct	36.4
Farm 1	Pen 2	32.5	No Ct	33.2	No Ct	No Ct	No Ct	31.3
Farm 1	Pen 3	32.8	No Ct	33.9	No Ct	No Ct	No Ct	39.4
Farm 2	Pen 1	35.3	No Ct	No Ct	37.6	No Ct	No Ct	31
Farm 2	Pen 2	32.8	No Ct	No Ct	No Ct	No Ct	No Ct	37
Farm 2	Pen 3	35.4	No Ct	No Ct	No Ct	No Ct	No Ct	32.8
Farm 2	Pen 4	34.8	No Ct	No Ct	No Ct	No Ct	No Ct	36.6
Farm 2	Pen 5	36.3	No Ct	No Ct	No Ct	No Ct	No Ct	42.4
Farm 3	Pen 1	34.4	No Ct	34.3	No Ct	No Ct	No Ct	31.3
Farm 3	Pen 2	34.9	No Ct	No Ct	No Ct	No Ct	No Ct	35.4
Farm 4	Pen 1	33.9	No Ct	34.3	No Ct	No Ct	No Ct	38
Farm 4	Pen 2	34.7	No Ct	32.8	No Ct	No Ct	No Ct	35.1
Farm 5	Pen 1	No Ct	No Ct	No Ct	37.8	No Ct	No Ct	42.9
Farm 5	Pen 2	No Ct	No Ct	36.8	No Ct	No Ct	No Ct	39
Farm 6	Pen 1	36	No Ct	35.2	No Ct	No Ct	No Ct	36.6
Farm 6	Pen 2	32.9	No Ct	35.4	No Ct	No Ct	No Ct	35.4
Farm 6	Pen 3	32.1	No Ct	33.7	37.4	No Ct	No Ct	33.3
Pluck Shop -1	N/A	28.4	No Ct	33.7	36.6	No Ct	No Ct	27.3
Pluck Shop -2	N/A	38.2	No Ct	No Ct	No Ct	29.9	No Ct	No Ct
+ve control	N/A	20.6	30.8	29	30.7	N/A	29.5	28.7
-ve control	N/A	No Ct	No Ct	No Ct	No Ct	No Ct	No Ct	No Ct

Ct: Cycle threshold; N/A: not available. Ct values ≤ 30 are strongly positive, Ct values between 31 and 35 are moderately positive, and Ct values > 35 are weakly positive.

**Table 2:** Combinations of multiple infections of *Eimeria* species detected in pens on six broiler farms and two ‘pluck shops’ in Trinidad, West Indies.

*Eimeria* Species Combinations	Pen Prevalence (n = 17)	No. of Farms/Pluck Shops
*E. acervulina* + *E. tenella*	5/17 (29.4%)	2 (Farm 2, Farm 3)
*E. mitis + E. tenella*	1/17 (5.9%)	1 (Farm 5)
*E. maxima + E. tenella*	1/17 (5.9%)	1 (Farm 5)
*E. acervulina + E. necatrix*	-	Pluck Shop 2
*E. acervulina* + *E. maxima + E. tenella*	8/17 (47%)	4 (Farm 1, Farm 3, Farm 4, Farm 6)
*E. acervulina + E. mitis* + *E. tenella*	1/17 (5.9%)	1 (Farm 2)
*E. acervulina* + *E. maxima* + *E. mitis + E. tenella*	1/17 (5.9%)	1 (Farm 6), Pluck Shop 1

Some farms had different combinations of *Eimeria* spp. infections within different pens.
